# Stakeholders’ Perceptions of Factors Influencing the Use of Take-Home-Naloxone

**DOI:** 10.3390/pharmacy8040232

**Published:** 2020-12-03

**Authors:** Taylor J. Holland, Jonathan Penm, Jacinta Johnson, Maria Sarantou, Betty B. Chaar

**Affiliations:** 1 Faculty of Medicine and Health, School of Pharmacy, The University of Sydney, Camperdown, NSW 2006, Australia; thol5537@uni.sydney.edu.au (T.J.H.); jonathan.penm@sydney.edu.au (J.P.); 2 Department of Pharmacy, Prince of Wales Hospital, Randwick, NSW 2031, Australia; 3 UniSA Clinical and Health Sciences, University of South Australia, Adelaide, SA 5000, Australia; Jacinta.Johnson@unisa.edu.au; 4 College of Medicine and Public Health, Flinders University, Bedford Park, SA 5042, Australia; sara0044@flinders.edu.au

**Keywords:** take-home naloxone, opioid, overdose, injecting drug users (IDUs), opioid antagonist, patients’ perspective, healthcare professional perspective

## Abstract

Background and Aims: Opioid associated death and overdose is a growing burden in societies all over the world. In recent years, legislative changes have increased access to naloxone in the take-home setting for use by patients with a substance use disorder and bystanders, to prevent opioid overdose deaths. However, few studies have explored the factors influencing the uptake by its multiple stakeholders. The aim of this scoping review was to explore the factors influencing the use of take-home naloxone from the perspectives of different stakeholders. Methods: A scoping review methodology was adopted with a systematic search of databases EMBASE, MEDLINE and PubMed. A variation of the search words “naloxone”, “opioid” and “overdose” were used in each database. The articles were screened according to the predetermined inclusion/exclusion criteria and categorized based on their key perspective or target population. Results: The initial database search yielded a total of 1483 articles. After a series of screening processes, 51 articles were included for analysis. Two key stakeholder perspectives emerged: patients and bystanders (n = 36), and healthcare professionals (n = 15). Within the patient and bystander group, a strong consensus arose that there were positive outcomes from increased access to take-home naloxone and relevant training programs. Despite these positive outcomes, some healthcare professionals were concerned that take-home naloxone would encourage high-risk opioid use. Conclusion: Take-home naloxone is slowly being introduced into community practice, with a sense of enthusiasm from patients and bystanders. There are still a number of barriers that need to be addressed from healthcare professionals’ perspective. Future research should be aimed at emergency care professionals outside of the US, who are most experienced with naloxone and its potential impact on the community.

## 1. Introduction

Opioid overdose and misuse is a significant public health burden worldwide and is a common cause of drug-related deaths in Australia [1]. In 2012 in Australia, there was a total number of 564 accidental deaths from opioid overdose [2], almost half that of total road accident associated deaths [3]. The number of opioid-related deaths has been sharply rising with a 21-fold increase observed in the state of Victoria, Australia, caused by oxycodone from 2000 to 2009 [4]. This has been, in part, due to a 15-fold increase in opioid prescriptions dispensed through the Australian Pharmaceutical Benefits Scheme (PBS) from 1992 to 2012 [5], with oxycodone being the main contributor, rising from 35.3 to 89.2 per 1000 population between March 2002 and August 2007 [6].

The increase in use of opioids has occurred for a variety of reasons. One large factor can be attributed to the increased acceptance of opioids for pain treatment, as they were once considered safe, with low risk of iatrogenic addiction [7]. However, recent trends show that rates of iatrogenic addiction and risks associated with prescribed opioids are higher than previously believed, when as early as 2001 the rate of hospitalization due to heroin was overtaken by non-heroin opioids [5,8]. Other factors contributing to this growth include more attempts to increase patient satisfaction scores, as well as strong marketing of opioids by the pharmaceutical industry [9,10,11].

Due to this increase in opioid use, there has also been an increase in opioid overdoses and opioid associated deaths, as mentioned above [6]. Research proposes that certain contributing factors such as low socioeconomic status, being of male gender, concurrent use of multiple medications, recent incarceration, homelessness and mental health conditions increase the likelihood of opioid overdose, from both prescription and illicit opioids [12,13,14]. This trend has not been limited to Australia but is being observed around the world with the United States (US) stating it is in an “opioid epidemic” [15].

With this increased number of accidental opioid-related deaths around the world, there has been a global shift to increase access to take-home naloxone for administration by patients and bystanders. Naloxone is a “rescue drug” that was approved for use by the US Food and Drug Administration (FDA) in 1971 for administration by emergency medical providers [16]. Naloxone is a mu-opioid receptor antagonist with the ability to reverse the effects of opioids on the central nervous system and improve acute respiratory status [17,18]. It is deliverable via injection or intranasal routes, with similar efficacy [19]. Patient preference and ease of administration in a non-hospital setting lean toward intramuscular or intranasal use; however, intranasal forms are not readily available in some countries, including Australia [19]. In small doses, naloxone is also indicated for reducing constipation associated with chronic opioid use; however, this is not the focus of this review [20].

Many countries around the world, including various states in the US, the United Kingdom, Canada, Italy and Australia have made naloxone available without a prescription, in the hope that those at risk of an opioid overdose, or their family and friends (bystanders), can easily have access to this life-saving medication [21,22,23,24]. The down-scheduling of naloxone occurred in Australia in February 2016, shifting it to Schedule Three (Pharmacist Only) medicine to be accessed without a prescription [25]. Prior to this, naloxone was available as a “Prescription-Only” medicine, primarily used in emergency medical service and hospital settings [25]. In 2011, a program named “Implementing Expanded Naloxone Availability in the Australian Capital Territory” (IENAACT) was commenced, trialling an increase in naloxone availability and awareness in the Australian Capital Territory (ACT) community [26]. This program ascertained that training participants (mostly opioid users) allowed successful administration of naloxone in an overdose situation, and that participants felt “positive emotional impacts” [26]. There were also 96 individual submissions to the Therapeutic Goods Administration (TGA), all reiterating that making naloxone “over the counter” will remove a barrier to its access, and that it is safe and holds no potential for misuse or abuse [23]. The successful outcomes of the IENAACT trial, the submissions to the TGA and a recommendation from The Advisory Committee on Medicines Scheduling (ACMS), all aided in the final decision to down-schedule naloxone [25].

Although policy changes such as this should markedly increase access to naloxone and its use in the community, the level of uptake and outcomes of take-home naloxone have not been clear. Furthermore, it is unknown if any specific challenges have arisen, which may be influencing the actions or opinions of all parties involved in take-home naloxone supply and use. Hence, the aim of this scoping review was to explore the perceived factors influencing the use of take-home naloxone from the perspectives of different stakeholders.

## 2. Materials and Methods

A scoping review methodology, following the Preferred Reporting Items for Systematic Reviews and Meta-Analyses (PRISMA) checklist, was adopted for this study in order to “describe in more detail the findings and range of research in particular areas of study, thereby providing a mechanism for summarizing and disseminating research findings to policy makers, practitioners and consumers” [27,28].

### 2.1. Search Strategy

The literature was searched and retrieved systematically from three databases: PubMed, EMBASE and MEDLINE on the 30 March 2017, in accordance with PRISMA guidelines. A variation of text words, key words and MeSH terms/subject headings were used: “*naloxone” AND “opioid analgesic” OR “opioid*” OR “opiate*” OR “narcotic*” OR “heroin” AND “intoxicat*” OR “drug overdos*” OR “overdos*”. See Table A1 for an example of the search strategies.

### 2.2. Study Selection

The study inclusion criteria included primary research articles published in the last ten years (2007–2017), because the injectable product for this purpose was introduced recently on the market, in peer reviewed journals focusing on stakeholders’ perceptions of factors affecting the uptake of take-home naloxone and its use anywhere around the world. Exclusion criteria were: language (not English) and studies focusing on pharmacologic mechanisms of actions, side effect profiles, dosage forms and naloxone in a non-take-home context, such as its use within a hospital. Articles that were not primary research and were excluded from selection included editorials, conference abstracts, notes, letters to the editor, reviews, case reports and supplements.

In the first phase of screening, duplicates, studies which were not primary research and those that did not meet our inclusion criteria based on the title, were removed. In the second phase of screening, abstracts were reviewed. Studies not in English were also excluded prior to the full text screening phase. Next, full texts were screened to focus on topics which met our inclusion criteria. Lastly, studies not identified by the database searches were sought by hand by searching the grey literature and the bibliographies of publications and were added to the pool of literature where relevant.

### 2.3. Assessment of Study Quality

The quality of included studies was assessed using the Joanna Briggs Institute (JBI) Critical Appraisal Checklists. The JBI Critical Appraisal Checklists utilized were specific to the various methodologies employed within the included studies. Within the checklists, each item was scored 0, 0.5 or 1, with higher scores indicating greater quality. As the checklists for various study methodologies contain between 8 and 13 items, scores were normalized to give a final score out of 100 to allow comparison across study types. Studies scoring above 75 were considered high quality, those scoring between 50–75 were considered medium quality and those scoring below 50 were considered low quality.

### 2.4. Data Extraction and Analysis

The following data were extracted and collated from the articles predominantly by one of the researchers (TH): country of study, data collection method, sample size, brief method and intervention details, main outcomes and funding source. The studies were then organized based on the sample population or perspectives. Studies were further analysed based on their study design, major findings and themes. Themes were then discussed and clarified (by TH, JP and BC) until a consensus was reached.

## 3. Results

**Figure 1:**
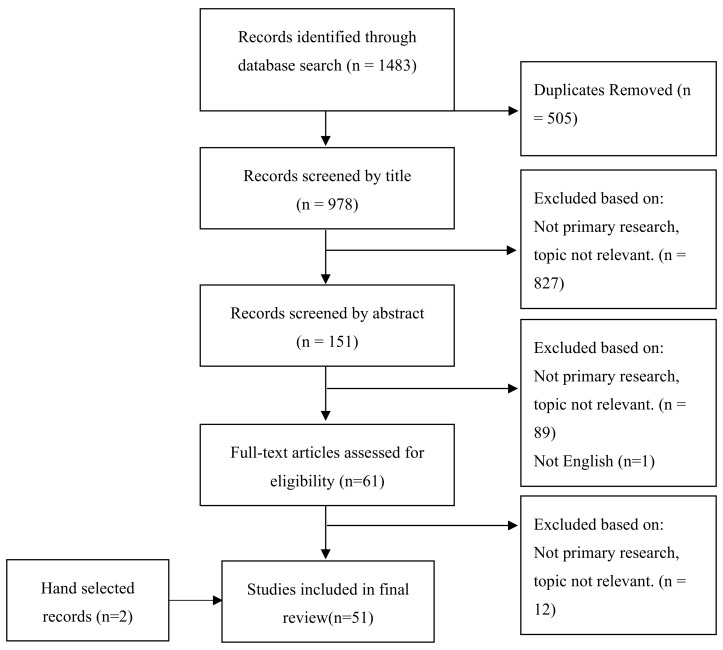
Preferred Reporting Items for Systematic Reviews and Meta-Analyses (PRISMA) Flow Diagram of Study Selection.

The search strategy generated 428 articles from MEDLINE, 474 from EMBASE and 581 from PubMed, yielding 1483 in total. After removal of duplicates, 978 were screened and through various phases of elimination 51 studies were included for analysis as shown in Figure 1. The mean study quality score was 76 (standard deviation ± 17) indicating most were of medium to high quality.

Studies were categorized into two groups pertaining to the perspective that each was based on. Thirty-six studies were found relating to the perspectives of patients likely to receive naloxone, and bystanders, who were members of the public who did not have an opioid addiction themselves, but were mostly friends and family of people who were at risk of opioid overdose. Of these, 64% were high quality, 25% medium quality and 11% low quality studies.

Fifteen studies were found exploring healthcare professionals’ perspectives, including various medical professionals, staff from prisons, needle exchange facilities and homeless shelter programs. In total, thirty-eight studies were conducted in the United States, seven in the United Kingdom, four in Canada, one in Norway and one in Australia. Of these, 60% were high quality and 40% were medium quality.

### 3.1. Patients’ and Bystanders’ Perspectives

The most prominent finding (24/36) within the patients’ and bystanders’ perspectives category was the positive outcomes resulting from access and training to take-home naloxone in terms of knowledge, confidence and rate of opioid reversals [29,30,31,32,33,34,35,36,37,38,39,40,41,42,43,44,45,46,47,48,49,50,51,52]. Four studies identified several facilitators including: the fact that naloxone is a life-saving measure; that it has the ability to empower people and potentially decrease drug use and that training was novel and interesting [53,54,55,56]. Some barriers identified included: the delivery of information from healthcare professionals as “professionally led health promotion initiatives appeared to lack credibility amongst the target population”, that administration can be challenging due to the potential need to titrate doses and the use of a needle in all routes of use other than intranasal administration. There was also fear of the unpleasant withdrawal symptoms that naloxone almost immediately precipitates, colloquially known as “dopesickness”. Furthermore, there was apprehension toward calling emergency services due to the fear of police interaction and the potential for incarceration [53,54,55,56].

In regard to the types of studies, pre-post training assessments was the most common (16/36) [30,32,33,34,35,37,40,41,43,44,45,48,49,52,57,58], followed by 11 cross-sectional surveys/questionnaires [29,31,36,39,42,50,59,60,61,62,63] and eight interviews [38,46,53,54,55,56,58,64]. There was one randomized control trial in the entire sample [51].

Needle exchange programs, homeless shelters, injecting drug user (IDU)-based surveys and other similar facilities were the main setting or source of recruitment for the studies (30/36) [29,30,31,32,33,34,35,36,38,39,40,41,42,43,44,45,46,47,48,49,50,52,53,54,55,56,60,62,63,64]. One study, conducted in an emergency department setting, which offered take-home naloxone to those deemed eligible, found that the majority of patients believed it was a “good idea” and that there is potential for emergency department based distribution to increase access to those most vulnerable to overdose [61].

### 3.2. Healthcare Professionals’ Perspectives

Identification and recognition of key facilitators and barriers to increasing naloxone access were the predominant themes of this category (11/15), unlike the patient and bystander results [65,66,67,68,69,70,71,72,73,74,75]. Seven of these studies were in the form of discussion groups and interviews, and the remaining four were surveys. Some of the facilitating aspects recognised were interventions that were “real-world” driven, provided education and training, had available resources and current involvement or awareness of other harm-reduction programs (such as opioid substitution therapy). Some of the perceived barriers included: financial and other logistical difficulties such as lack of staff and time to appropriately train and educate patients and bystanders, regulations and legalities and lack of education and training. Examples of specific patient-related barriers included concerns regarding offending patients who had not previously experienced an overdose in response to the offer of take-home naloxone as well as a stigma. Stigma is multifaceted, including patients being discriminated against by peers for having this medication, and is related to some healthcare professionals’ expressing lowered motivation and interest toward helping people who are using opioids. Two pre-post evaluation studies showed positive outcomes from healthcare professionals receiving additional training for the use of take-home naloxone [76,77].

Two studies were associated with a needle exchange clinic or safe injecting room types of settings [74,77]. Four studies were conducted from a pharmacy perspective, with two of them identifying similar facilitators and barriers as mentioned above [65,67] and two showing mostly positive attitudes of pharmacists toward take-home naloxone but also highlighting a lack in knowledge [71,75]. Emergency care provider opinions were the focus of two studies, one concluding a predominantly negative attitude toward take-home naloxone with the opinion that it would not decrease death rates [73]. The second identified several facilitators and barriers as mentioned above [67].

## 4. Discussion

This review has explored the literature available in regard to factors influencing the use of take-home naloxone from the perspectives of patients/bystanders and healthcare professionals. From the perspectives of patients and bystanders, the findings in the literature depicted positive responses from the increased access to take-home naloxone [29,30,31,32,33,34,35,36,37,38,39,40,41,42,43,44,45,46,47,48,49,50,51,52]. A sense of empowerment increase in confidence and ability to recognize overdose symptoms were just some of the encouraging conclusions that these studies made. In conjunction with these findings, it was clear that naloxone administrations were successful in reducing opioid-related overdose deaths, which is ultimately the goal of all harm minimization interventions [30,32,33,34,35,38,39,41,42,43,44,45,46,47,48,50,52].

In 1985, the Australian Government adopted harm minimization as a national framework in an attempt to address the range of drug and alcohol issues in society [78]. Whilst it is clear that take-home naloxone has support from its potential users in the community as a harm minimization project, healthcare professionals have expressed concerns about its uptake, as it was perceived to encourage high-risk opioid use [66,68,69,70,72,73,74,75]. Similar concerns were, and still are, expressed in regard to needle exchange and distribution programs [79]. Needle exchange programs have been implemented in Australia since the mid-1980s under the harm minimization framework mentioned above [79]. Although there has been no evidence that take-home naloxone or syringe exchange programs increased drug use, this stigma still remains [80,81,82]. In fact, studies have shown that naloxone has the potential to decrease drug use, as having access to naloxone motivated and empowered patients to be more health conscious [33,45].

To highlight the stigma associated with naloxone, a comparison can be drawn with adrenaline for anaphylaxis. Both naloxone and adrenaline are patient administered rescue medications that save lives; however, the introduction of adrenaline induced no resistance from the community compared to other harm minimization programs such as syringe exchange or methadone [83]. A systematic review around healthcare professionals’ perspectives showed that they expressed “lowered regard, less motivation and feelings of dissatisfaction” toward patients with substance use disorders, consolidating this notion of stigma [84]. As mentioned, naloxone is just as much a life-saving medication as adrenaline, and a healthcare professional’s decision to withhold it from patients based on this stigma is a violation of all principles of professional ethics in healthcare [85,86]. Codes of ethics state that, despite a conscientious objection to the supply or prescribing of a medical product, healthcare professionals have an obligation to place the best interests of the patient above all else and, at the very least, maintain continuity of care to all patients [85,86].

It is also known that illicit use of opioid medication, prescription or not, is not the only cause of opioid overdose [87]. Chronic pain patients are also at risk of opioid overdoses due to pharmacokinetic changes with age or confusion about dosing or instructions of use [88]. Despite this, current studies are strongly focused on injecting drug users and patients involved with needle exchange programs, homeless shelters and similar facilities. This disproportionate focus may be due to the fact that talking to non-illicit opioid users about take-home naloxone was identified as a barrier by many healthcare professionals’ due to the fear of offending them [67,68]. A way to mitigate this risk would be to educate healthcare professionals on how to identify “high risk” chronic opioid using patients for the potential of opioid overdose and provide all of these patients with take-home naloxone [89,90].

Many healthcare professionals also emphasized the lack of education and training on take-home naloxone in this review [67,68,69,70,71,72,73,75]. Although codes of ethics in healthcare also state that healthcare professionals are bound by an obligation to be life-long learners, it is clear that patients are being adversely affected by healthcare professionals’ lack of knowledge [85,86]. Two studies showed that training lasting around an hour was sufficient to increase the knowledge of homeless shelter staff and other healthcare providers [76,77]. All healthcare professionals who prescribe opioids or care for patients at risk of opioid overdose should be provided with training on take-home naloxone.

Barriers identified in this review have all been encountered previously by other harm prevention strategies such as the methadone substitution therapy. Methadone programs were introduced in Australia in the 1970s and over time have slowly overcome barriers associated with training, education and stigma, similar to those identified by the professionals’ perspectives category in this review [91,92]. A systematic review about stigma among healthcare professionals towards patients with substance use disorder stressed the importance of training of healthcare professionals “in order to extend the knowledge, skills and self-efficacy of professionals working with patients with substance use disorders” [84]. Alongside this training, two factors were identified by McArther 1999 that assisted methadone in gaining traction in communities and overcoming these barriers including the high demand for it from drug users themselves, and the eventual realization that it played a role in reducing crime rates and reducing HIV/AIDs transmission [92]. This last point indicates that with time, naloxone could gain community awareness, proving its worth and benefits in a take-home setting, as was the case for methadone. In fact, an article in 2007 by Beletsky et al. supported this notion and concluded that physicians with more experience and awareness of patients with substance use disorders were more inclined to respond positively to take-home naloxone prescriptions [93].

Emergency care providers were highlighted in this review as they hold expertise in opioid-related overdoses and take-home naloxone has the potential to impact the nature of their interactions with overdosing patients [67,73]. However, emergency providers were found to hold negative views towards patients with substance use disorders, with one study showing that more than half viewed take-home naloxone training as an ineffective strategy to reduce opioid-related deaths [73]. Addressing emergency providers’ concerns and obtaining their support is crucial for successful uptake of take-home naloxone. In addition to the large number of patients that present to the emergency department (ED) that are at risk of an opioid overdose, evidence also shows that patients are likely to stay on the medications prescribed for them in hospital [94]. With this trend in mind, it is imperative that emergency physicians are the focus point for further education about take-home naloxone, in order to increase the dissemination of this life-saving medication into the community. Once the initial uptake has been established, it is assumed that other healthcare providers such as general practitioners and pharmacists are likely to follow this pattern of distribution.

The perspective of the pharmacist was also explored in this review [66,68,72,76]. As take-home naloxone no longer requires a prescription in many countries, pharmacists are becoming increasingly involved in its distribution. Pharmacists are arguably the most accessible healthcare professionals and may be the first point of contact patients have with the healthcare system [72]. Two studies communicated policy regulations as a large barrier to take-home naloxone from a pharmacist’s perspective. Regulatory issues are particularly prevalent in the US, where legislation differs between states, causing confusion for all parties involved, in relation to the varying degrees of access to take-home naloxone [95,96]. Furthermore, although pharmacists were found to express positive attitudes towards harm-reduction services, very few stocked naloxone and the majority lacked confidence in their ability to educate patients on naloxone use [72]. Another study noted that pharmacists were supportive of take-home naloxone, but were unaware of the high prevalence of opioid overdose [76]. This limited cognizance regarding take-home naloxone and the opioid burden in general is reflected in the lack of uptake of this medicine in the community. The paucity of information surrounding the role of pharmacists and emergency care professionals, two key stakeholders in the future of take-home naloxone, is an area necessitating further research.

Limitations: The current findings need to be considered in light of several limitations. The review was limited to articles in English; however, there was only one study identified that was not in English, thus it is unlikely that this restriction greatly impacted the results [96]. Second, the majority of the studies included were from the US, limiting the applicability or generalizability of these results to a worldwide setting. Third, only three databases were used for the systematic search strategy; however, this included two of the largest, most comprehensive databases in this area of research. Future researchers could conduct a similar search in databases such as Cumulative Index to Nursing and Allied Health Literature (CINAHL) and International Pharmaceutical Abstracts (IPAs), in order to gain additional literature from a broader range of medical professions. Despite this, we believe that this search was sufficient to support our findings and that all key themes and perspectives were identified.

## 5. Conclusions

Findings of this study indicated that patients and bystanders who may use take-home naloxone were eager and have positive attitudes towards its use; however, there remain some barriers from a healthcare professional’s perspective. In particular, it was found that stigma around drug use negatively affects the implementation/uptake of take-home naloxone, as some healthcare professionals appear to view these patients. Future research should be aimed at exploring how to gain stronger support from emergency care professionals who are experienced with opioid overdoses and the potential impact of take-home naloxone on the community. The obstacles identified in this study, regarding the implementation of take-home naloxone, were not new concepts. With time, allowing for the efficacy of naloxone to be proven, and with an increase in education and training for all parties involved, more patients at risk of opioid overdose should see access to this life-saving medication. In addition, the impact of take-home naloxone should be explored more extensively in settings outside of the US.

## Appendix A

**Table A1:** Example search strategy from medline.

#	Search Statement	Results
1	* naloxone/	20,831
2	opiate/	79,558
3	narcotic agent/	6672
4	heroin.tw.	16,875
5	2 or 3 or 4	107,585
6	intoxication/	216,931
7	drug overdose/	20,940
8	6 or 7	230,238
9	1 and 5 and 8	544
10	limit 9 to human	474

* The asterix in this context is what is known as a “wildcard” and represents any group of characters, including no character.

**Table A2:** Summary of Included Studies from Patients’ and Bystanders’ Perspectives.

Patients’ and Bystanders’ Perspectives						
Author, Title and Journal	Country	Data Collection Method	Participants	Interventions/Study Details	Outcomes	Funding/ Sponsorship
Bachhuber et al., 2015 Messaging to increase public support for naloxone distribution policies in the United States: results from a randomized survey experiment. PLOS One [58]	US	Cross-sectional survey post-randomized exposure to intervention	N = 267 Control (C) N = 260 Factual (F) N = 266 Factual + Refutation (FR) N = 264 Sympathetic narrative (SN) N = 276 Sympathetic + Factual (SF) 50.1% women, 73.5% white, 22% 55–64 years old	Random selection of US households from GfK survey research panel were exposed to three different types of persuasive messages for support of naloxone alone or in combination (or compared too no exposure) and support for naloxone distribution polices was assessed	- Support from C was strongest for training first responders (63.2%) and passing laws to protect people if they call for medical help (52.4%) - 77.4% of F group supported training first responders and 80.5% of SN group - 58% of F and 55.6% of SN groups supported passing laws to protect those calling for medical help - 45.5% of F group supported providing naloxone to family members and 40.1% of the SN group - 73.7% of F, 77% of SN believed naloxone would save lives (60% of control) - SF group had high support for all outcomes	American International Group Inc
Bagley et al., 2015 Overdose education and naloxone rescue kits for family members of individuals who use opioids: characteristics, motivations, and naloxone use. Substance Abuse [29]	US	Cross-sectional survey	N = 126 family members of opioid users	A convenience sample of community support group attendees was surveyed The community group offered naloxone training lasting 20 min. Compared those who received overdose education and naloxone to those who did not.	-Those who received training were more likely to be a parent (91% versus 65%) and have daily contact with opioid user (54% versus 33%) - 60% felt encouraged from education at meetings, 72% wanted naloxone in the house - Trainees endorsed “greater sense of security” and “improved confidence” - Of the 27 untrained subjects, 2 reported it was not necessary for training and 2 reported not living with the user	National Institute on Drug Abuse
Barocas et al., 2015 High uptake of naloxone-based overdose prevention training among previously incarcerated syringe-exchange program participants. Drug and Alcohol Dependence [59]	US	Cross-sectional survey	N = 309 people who use drugs with no history of incarceration N = 234 people who use drugs with history of incarceration Mean age 31.6, 79% white, 69% male	Participants were injecting drug users from Lifepoint Needle Exchange program	- 198/234 (85%) had observed and 96/234 (41%) had experienced an overdose compared to 174/309 (56%) and 70/309 (23%) of the group who had no history of incarceration - 162/234 (69%) of the history of incarceration group had been trained to administer naloxone compared to 175/309 (57%) of the non-incarcerated groupand 98/234 (46%) had administered naloxone compared to 85/309 (37%)	Clinical and Translational Science Award
Bennett et al., 2011 Characteristics of an overdose prevention, response, and naloxone distribution program in Pittsburgh and Allegheny County, Pennsylvania. Journal of Urban Health: Bulletin of the New York Academy of Medicine [30]	US	Pre-post survey T_0_: Baseline before training T_R_: Returning for refill	N = 426 people who use drugs trained in naloxone use	Training (25 min) on prevention, identification and response to an overdose and how to administer naloxone, rescue breathing and calling 911 Volunteer physicians prescribed naloxone	T_0_: 170/426 (40%) had overdosed in their life - EMS called 33% of times T_R_: 89/141 (63%) reported using naloxone - EMS called 10% of times - 85/89 (96%) reported positive outcomes after naloxone administration - 61% performed rescue breathing they were taught in training	National Institute of Drug Abuse
Bird et al., 2016 Effectiveness of Scotland’s National Naloxone Programme for reducing opioid-related deaths: a before (2006–10) versus after (2011–13) comparison. Addiction [31]	UK	Cross-sectional record analysis; two time points investigated	N = 193 opioid-related deaths in 2006–10 within 4 weeks of prison release N = 76 opioid-related deaths in 2011–12 within 4 weeks of prison release	The national naloxone programme provides free naloxone and training to all those at risk of opioid overdose, including prisoners at liberation National Records of Scotland release official statistics on the number of drug-related deaths	- Reduction in prison release opioid-related death by 3.5% (9.8%, 193/1970, versus 6.3%, 76/1212) - Distribution of 11,898 naloxone kits assumed to have prevented 42 prison release opioid-related deaths	Medical Research Council
Delaney et al., 2016 Coprescribing naloxone for patients on chronic opioid therapy: lessons learned from a patient-safety initiative in primary care training sites. Journal of Opioid Management [56]	US	Pre-post questionnaire: T_0_: Baseline naloxone prescription through medical records T_P_: Obtaining a prescription	N = 204 people who use opioids	Resident physicians initiate patient selection regarding naloxone prescription Participants were taught about naloxone, first by the resident but part way through the study changed to the pharmacist	T_0_: No co-prescriptions of naloxone written T_P_: 53/204 (25%) of patients were written prescriptions - Patients who accepted the prescription often had been exposed to an overdose	None stated
Doe-simkins et al., 2014 Overdose rescues by trained and untrained participants and change in opioid use among substance-using participants in overdose education and naloxone distribution programs: a retrospective cohort study. BMC Public Health [32]	US	Retrospective cohort study. post-questionnaire: T_0_: Baseline demographics T_R_: Returning for refill	N = 295 trained N = 78 untrained people who use drugs	Training (5–60 min) included overdose prevention, assessing for overdose, seeking help, rescue breathing, naloxone administration, post-administration support Using data from Massachusetts Opioid Overdose Prevention Pilot Program (restricted from September 18, 2006 to December 31 2010)	T_R_: No significant differences in the overdose event if the rescuer was previously trained or not - Naloxone was used successfully 295/303 (97%)	Centers for Disease Control and Prevention
Dong et al., (2012) Community-based naloxone: a Canadian pilot program. Canadian Journal of Addiction Medicine [33]	Canada	Pre-post questionnaire: T_0_: Baseline before training T_a_: Immediately after training T_1_: 1 year after training	N = 50 clients, Streetworks needle exchange program	Individual training (30–40 min) focused on harm reduction, including overdose prevention, recognition of overdose, provision of respiration, naloxone education and IM administration and calling EMS. Optional CPR was offered	T_0_: 39/50 (78%) had experienced an overdose and 46/50 (92%) had witnessed an overdose T_a_: Naloxone use reported 9 times, all successful outcomes - 9/9 used a clean syringe - 8/9 alcohol swabbed the skin before injection - 1/9 EMS contacted - 1/9 participant felt unsure what to do T_1_: 15/15 found training worthwhile - 11/15 reported decrease in drug use	Health Canada
Enteen et al., 2010 Overdose prevention and naloxone prescription for opioid users in San Francisco. Journal of Urban Health [34]	US	Pre-post questionnaire: T_a_: immediately after training Interviews or questionnaire: T_r_: receiving naloxone refills	N = 470 people who use drugs received a naloxone refill	Training was 10–30 min focusing on overdose identification, revival, calling EMS and administering naloxone Physicians prescribed and dispensed naloxone	T_r_: 215/470 naloxone supplies were used in overdose and 311/470 were reported lost - 357/399 overdose events were successful - 116/399 reported calling EMS and 123/399 reported using an additional strategy they were taught	None stated
Galea et al., 2006 Provision of naloxone to injection drug users as an overdose prevention strategy: early evidence from a pilot study in New York City. Addictive Behaviors [22]	US	Pre-post questionnaire: T_0_: Baseline before training T_3m_: 3 months after training	N = 25 needle exchange program clients	Training focused on assessing overdose victim, calling EMS, positioning and rescue breathing and administration of up to two doses of naloxone IM over one hour Conducted in small groups or individually Physician met with participants to distribute naloxone	T_0_: 13/25 experienced and 19/25 had witnessed an overdose - 11/19 (58%) called EMS T_3m_: 9/11 (82%) called EMS - 10/17 naloxone administrations for overdose, all 10 successful - 15/20 (75%) felt comfortable using naloxone - Increase in appropriate response techniques (placing in rescue position from 5.3% to 18.3%)	Tides Foundation
Green et al., 2008 Distinguishing signs of opioid overdose and indication for naloxone: an evaluation of six overdose training and naloxone distribution programs in the United States. Addiction [35]	US	Cross-sectional survey	N = 30 trained N = 32 untrained people who have or currently use drugs 72.6% male, 45.8% reporting previous overdose	Staff from six overdose training and naloxone distribution sites with similar training programs agreed to participate Staff from each site selected 5 previously trained clients and 5 untrained Participants assessed 16 cases about the nature of the overdose	- Knowledge of opioid overdose higher among trained 85.2% versus 68.3% (*p* < 0.005) - Similar indecision recognizing non-overdose and non-opioid overdose (*p* = 0.42) - Trained participants were more likely to have responded to an OD - Medical experts assigned approximately 10% more codes to the “don’t know/unsure/not enough information” category, compared to trained respondents	National Institute of Mental Health
Jones et al., 2014 Brief overdose education can significantly increase accurate recognition of opioid overdose among heroin users. International Journal of Drug Policy [36]	US	Pre-post assessment: T_0_: Baseline before training T_a_: Immediately after training	N = 44 trained N = 40 untrained people who use heroin	Semi-structured lecture focused on risk factors, signs and response for overdose lasting 13–18 min Control group did not receive training	T_0_: Equivalent demographic breakdown between the groups - Assessment and confidence scores did not differ between groups T_a_: Increase in trained groups increase ability to identify opioid-overdose (*p* < 0.05) - Trained increase confidence from 7.9 to 9.4 (*p* < 0.05)	National Institute on Drug Abuse grant
Kestler et al., 2017 Factors associated with participation in an emergency department-based take-home naloxone program for at-risk opioid users. Annals of Emergency Medicine [60]	Canada	Cross-sectional survey	N = 201 emergency department attendees 37% women, 26% indigenous	Participants recruited from inner-city teaching and referral center if they were over 16 and high risk of opioid overdose Five-minute training followed by 15–20 min questionnaire, and naloxone supply	- 183/200 (91.5%) thought take-home naloxone was a good idea and 168/200 (84%) thought ED was a suitable location - 137/201 (68.2) accepted naloxone kit and brief training	None stated
Lankenau et al., 2013 Injection drug users trained by overdose prevention programs: responses to witnessed overdoses. Journal of Community Health [37]	US	Interviews	N = 30 people who inject drugs Majority male, non-white and homeless	Participants from homeless shelters, trained in naloxone use were recruited Series of open and closed ended questions	- Most participants first response was to stimulate the victim - 911 was often called but with fear of arrest - CPR and rescue breathing was administered - Half injected naloxone, feeling capable and with few reported difficulties	National Institutes of Health
Leece et al., 2013 Development and implementation of an opioid overdose prevention and response program in Toronto, Ontario. Canadian Journal of Public Health [38]	Canada	Cross-sectional survey	N = 20 people who use opioids, trained in naloxone use	Participants recruited by word of mouth and flyers Training was 20 min exploring prevention and response to overdose, including calling EMS, chest compression IM naloxone administration and post-overdose care	T_a_: Sense of empowerment after completing the training - 17 naloxone administrations post-training, all successful	None stated
Lott and Rhodes 2016 Opioid overdose and naloxone education in a substance use disorder treatment program. American Journal on Addictions [39]	US	Pre-post questionnaire: T_0_: Baseline before training T_a_: Immediately after training (training group only) T_3m_: 3-month follow up	N = 14 control N = 43 training people who use drugs	Control group received information about where to obtain naloxone overdose kits Training session was a 30–45 min lecture, detailing signs of overdose and naloxone use	T_0_: 4/57 possessed naloxone in the past T_a_: Improvement in knowledge from 32.6 to 39.1 (*p* < 0.0001) after training T_3m_: Knowledge maintained in trained group 38.4 (*p* < 0.0001) - Control group also showed improvement (32.7 to 36.3 (*p* < 0.005)	Linden Oaks
Madah-Amiri et al., 2017 Rapid widespread distribution of intranasal naloxone for overdose prevention. Drug and Alcohol Dependence [40]	Norway	Pre-post questionnaire (optional): T_0_: Baseline before training T_R_: Receiving naloxone refill	N = 1322 nasal naloxone sprays distributed to people who use drugs	Training was brief and flexible individually or in small groups Training looked at naloxone administration, assembling the device, titrating the dose and administration, rescue breathing and CPR, aftercare, side effects and withdrawal symptoms	T_0_: 366/433 demonstrated risks factors for overdose - 361/394 (92%) who witnessed an overdose called EMS T_R_: 743/1322 returned for refill - 401/743 completed questionnaire - 277/401 (70%) used supply on an overdose, 265/277 (96%) the victim survived - 260/277 (94%) used actions taught - 183/277 (66%) called EMS - “No adverse effects” was the most common answer for side effects 76/277 (27%)	Norwegian Directorate of Health
Maxwell et al., 2006 Prescribing naloxone to actively injecting heroin users: a program to reduce heroin overdose deaths. Journal of Addictive Diseases [41]	US	Cross-sectional description of service data	N = More than 3500 multi-dose vials of naloxone were distributed	Education on basic opioid actions and overdose, risk factors and prevention techniques Physician supplies naloxone	- To date 319 peer overdose reversals - One unsuccessful revival has been reported; however, it was thought to be attributed to other factors - In 5 instances, victim did not respond until a second injection of naloxone was given	None stated
McAuley et al., 2016 Engagement in a national naloxone programme among people who inject drugs. Drug Alcohol Dependence [61]	UK	Cross-sectional survey pre- and post-implementation of a national programme	Approximately N = 2000–3000 drug users each year	Naloxone specific questions from 2 Needle Exchange Surveillance Initiative (NESI) surveys were analysed	- Participant prescribed naloxone increased from 175/2146 (8%) in 2011–2012 to 745/2331 (32%) in 2013–2014 - Participants who carried naloxone on them every day decreased from 27/169 (16%) in 2011–2012 to 39/471 (5%) in 2013–2014	Scottish Government
Mueller et al., 2017 Attitudes toward naloxone prescribing in clinical settings: a qualitative study of patients prescribed high dose opioids for chronic non-cancer pain. Journal of General Internal Medicine [52]	US	Interviews	N = 24 chronic opioid users completed interviews Mean age 53.9, 66.6% females, average length of opioid prescription 11.2 years	Patients who received three or more opioid prescriptions within 90-day period were recruited A priori template of codes was used as a guide to code the interviews	- Barriers to naloxone: limited prior education and knowledge, low perception of overdose risk, fear of exacerbating providers’ concerns about opioid misuse and of consequences and loss of pain treatment if naloxone is used - Facilitators to naloxone: recognition of the utility to prevent overdose death, providers who engaged in empowering and non-judgmental communication practices, naloxone training and education	National Institute on Drug Abuse of the National Institutes of Health
Oliva et al., 2016 Patient perspectives on an opioid overdose education and naloxone distribution program in the U.S. Department of Veterans Affairs. Substance Abuse [53]	US	Focus groups	N = 21 substance use disorder participants	Training was 1 h with slideshow, videos and handouts, addressing epidemiology of overdose deaths, how to prevent, recognize and respond to an overdose including rescue breathing, naloxone administration and calling EMS	- Benefits—OEND training is interesting and novel, the potential to save lives and empower people - Concerns—challenges using syringes (IM kit) - Differing opinions—naloxone kits may contribute to relapse, legal and liability issues, challenges in involving a family in OEND training, uncertainty about uses/effects of naloxone	VA Health Services and Development’s Quality Enhancement Research Initiative
Piper et al., 2008 Evaluation of a naloxone distribution and administration program in New York City. Substance Use and Misuse [42]	US	Post questionnaire: T_R_: Returning for a refill	N = 122 people who use opioids	Training program— Skills and Knowledge on Overdose Prevention (SKOOP), focused on overdose prevention education, naloxone administration and the importance of educating family Physicians provided a naloxone kit	T_R_:71/122 (58.2%) witnessed an overdose since training, and 50/71 (70.4%) used naloxone - In total naloxone was administered 82 times, 68 (83%) known to live and 14 (17.1%) unknown outcomes - 97/122 (82.2%) felt comfortable using naloxone	New York City Council
Rowe et al., 2015 Predictors of participant engagement and naloxone utilization in a community-based naloxone distribution program. Addiction [43]	US	Pre-post questionnaire (optional): T_0_: Baseline before training T_R_: Receiving naloxone refill	N = 2500 participants who might witness or experience an overdose	Analysis of data from DOPE (Drug Overdose Prevention Education Project) which included 5–10 min training and naloxone kit distribution	T_R_: 702 naloxone administrations post-training - 192/702 (27.4%) also contacted EMS - 673/702 (95.7%) known to survive after naloxone reversal	National Institutes of Health
Rowe et al., 2016 Neighborhood-level and spatial characteristics associated with lay naloxone reversal events and opioid overdose deaths. Journal of Urban Health [62]	US	Cross-sectional	N = 316 overdose reversals	Locations of all opioid overdose deaths in San Francisco from 2010 to 2012 were extracted Naloxone administration events from 2010 to 2012 extracted from the DOPE project data Census tract Socioeconomic status was measured by tract median income and economic inequality	- 44/195 (23%) areas analysed had DOPE project distribution site - Distance to nearest DOPE site was not statistically significantly associated with number of overdose deaths (*p* = 0.093) - Distance to the nearest DOPE site (up to 4000 m) was independently associated with a 49% lower count of naloxone reversals *p* = 0.001) - The mean number of reversal events declined across increasing quartiles of distance to nearest DOPE site and median income increased across quartiles of number of drug arrests, percentage black or African American residents, and population density	National Institutes of Health
Seal et al., 2003 Attitudes about prescribing take-home naloxone to injection drug users for the management of heroin overdose: a survey of street-recruited injectors in the San Francisco Bay Area. Journal of Urban Health: Bulletin of the New York Academy of Medicine [63]	US	Qualitative interviews (themes analysed quantitatively)	N = 82 people who inject drugs 36% female, 57% African American, 31% white, 46 median age and 27 years was median duration of injection	Participants were invited from the Urban Health Study (a semi-annual study to survey HIV and hepatitis) based on their report of heroin overdose	- 87% would participate in a THN training program - 35% believed that having naloxone would make them feel more comfortable to use more heroin, the majority did not feel this way - 62% responded they might be less likely to call EMS if they administer naloxone	Open Society Institute
Seal et al., 2005 Naloxone distribution and cardiopulmonary resuscitation training for injection drug users to prevent heroin overdose death: a pilot intervention study. Journal of Urban Health [44]	US	Post interview: T_1-6m_: Monthly for 6 months post-training	N = 24 people who inject drugs	Training involved four, two-hour interactive sessions. 1: recognize overdose and overdose prevention 2: CPR practice 3: accessing medical services 4: naloxone administration Naloxone kit was dispensed after completion of training	T_1-6m_: 20 overdoses were witnessed, naloxone was used in 15 cases and there was some form of other intervention in all 20 cases - EMS was contacted in 6/20 cases - Reasons for not contacting was fear of police in 10 cases, no phone in 5 cases and no perceived need in 5 cases T_6m_: Knowledge of overdose had increased -Frequency of injecting heroin decreased with people injecting 90+ times in a month from 11/24 to 0/24 (*p* = 0.003)	None stated
Sherman et al., 2008 A qualitative study of overdose responses among Chicago IDUs. Harm Reduction Journal [45]	US	Qualitative interviews	N = 31 injecting drug users 81% male, median age 38, 81% injected opioids daily	Semi-structured qualitative interviews with participants recommended by needle exchange staff	- All cases of naloxone use were successful - Participants were comfortable and confident - Unanimous fear of contacting 911 due to fear of police involvement	Tides Foundation
Strang et al., 2008 Overdose training and take-home naloxone for opiate users: prospective cohort study of impact on knowledge and attitudes and subsequent management of overdoses. Addiction [46]	UK	Pre-post questionnaire: T_0_: Baseline before training T_a_: Immediately after training Interview T_2m_: 2 months	N = 239 opiate users	Training focused on risk factors, recognition and actions to be taken in an overdose, including naloxone administration Participants were provided with naloxone	T_0_: Overall knowledge of overdose mean score was 16.7/26 and at T_a_:21.4/26 (*p* < 0.001) - 151/196 (77%) willing to administer naloxone and at T_a_: increase to 192/194 (99%) T_2m_: 169/173 (98%) reported confidence in ability to recognize opiate overdose and 168/172 (97%) confident in their ability to manage it - 11 naloxone administrations - No formal adverse events reported	National Treatment Agency for Substance Misuse
Strang et al., 1999 Preventing opiate overdose fatalities with take-home naloxone: Pre-launch study of possible impact and acceptability. Addiction [57]	UK	Interviews analysed quantitatively	N = 312 community sample (people who inject drugs) Mean age 30.6, age first injected 20.1, 37.2% women N = 142 treatment sample (people who inject drugs) Mean age 35.8, age first injected 19.5, 26.8% women	Community sample (control) and treatment sample (Methadone participants) Treatment sample interviews were conducted after community sample analysis in order to address any topics brought up by the community sample	- 49/142 (35%) had heard of naloxone - 90/142 (70%) of treatment group considered naloxone distribution a good idea - 9/142 (6%) reported it may increase their heroin dose - Estimated that 2/3 of the 69 overdose fatalities reported by participants could have bene prevented	None stated
Tobin et al., 2009 Evaluation of the staying alive programme: training injection drug users to properly administer naloxone and save lives. International Journal of Drug Policy [72]	US	Pre-post questionnaire: T_0_: Baseline before training T_6m_: 6-month follow up	N = 250 people with opioid addiction	Training details were not discussed	T_0_: No one had used naloxone T_6m_: 19 reported using naloxone - Knowledge improved for 46% of the sample about risk of relapse after naloxone, 35% did not know at either time point or knowledge decreased - 25% reported increase comfort in managing overdose post-training and 32% were negative or unsure at both time points	National Institute on Drug Use
Wagner et al., 2010 Evaluation of an overdose prevention and response training programme for injection drug users in the skid row area of Los Angeles, CA. International Journal of Drug Policy [48]	US	Pre-post questionnaire: T_0_: Baseline before training T_3m_: 3-month follow up	N = 66 people who inject drugs	Training was individual or in small groups for 1 h addressing opioid overdose mechanism, prevention, recognition and response Physician dispensed naloxone after training completion	T_3m_: Overall knowledge increased from 77%–92% (*p* < 0.0001) - 53% reported decrease in drug use - No significant change in attitudes about overdose response - Number of recommended response techniques increased from 2 to 3.3 (*p* = 0.01)	“foundation support and private fundraising”
Walley et al., 2013 Opioid overdose rates and implementation of overdose education and nasal naloxone distribution in Massachusetts: interrupted time series analysis. BMJ [49]	US	Cross-sectional survey	N = 4857 people who inject drugs or bystanders enrolled in naloxone training	Trainings from 10–60 min about risk factors of overdose and naloxone administration Baseline and refill questionnaires were obtained from the Massachusetts Department of Public Health OEND program (2006–2009)	- 327 rescue attempts 150/153 (98%) used naloxone successfully - Decrease in opioid-related deaths in communities with OEND	Center for Disease Control and Prevention
Williams et al., 2014 Training family members to manage heroin overdose and administer naloxone: randomized trial of effects on knowledge and attitudes. Addiction [50]	UK	RCT Assessment: T_a_: Immediately after training T_3m_: 3 months post-training	N = 87 experimental group N = 75 control family members and carers	Opioid overdose knowledge/attitude scale (OOKS and OOAS) were developed Training (60 minute) on overdose risk factors, recognition, actions to take, what naloxone is and how to use it Control group did not receive training	- T_3m_: Control group had an 11% increase in knowledge and 35% increase for training - Control group had a 20% increase in positive attitude scored and 54% for training group.	Alban Programme and Institute of Social Psychiatry and University of London
Worthington et al., 2006 Opiate users’ knowledge about overdose prevention and naloxone in New York City: a focus group study. Harm Reduction Journal [54]	US	Focus groups	N = 8 people who use opioids 1st focus group 37% African American females, 37% white males N = 5 people who use opioids in the 2nd focus group 80% white males	Recruited by flyers, word of mouth etc, from Lower East Side Harm Reduction Center (LESHR) discussing overdose experience especially with naloxone and comfort with naloxone	- Both groups expressed some hesitation about take-home naloxone - 4 major themes addressed: support for naloxone as a lifesaving measure, challenges of administering naloxone during an overdose, fear of dopesickness and police arrest	Tides Foundation
Wright et al., 2006 Homeless drug users’ awareness and risk perception of peer “Take Home Naloxone” use—a qualitative study. Substance Abuse Treatment, Prevention, and Policy [55]	UK	Qualitative interviews	N = 27 people who use heroin 19 men and 8 women	Participants recruited from 3 sites who deliver services to homeless people who use drugs	- Participants had good prior knowledge about naloxone; however, many mistook it for adrenaline - Clear willingness to administer THN if required - Concerns about misuse and malicious use - Participants often saw it as obviating the need to call EMS - Some participants revealed that current information from professionals is often not appealing or likely to increase their knowledge, other saw a role for professionals in education	None stated
Yorkell et al., 2011 Opioid overdose prevention and naloxone distribution in Rhode Island. Medicine and Health, Rhode Island [51]	US	Pre-post report about use: T_3m_: 3-month follow up OR after 1^st^ naloxone use (whichever comes first)	N = 120 people who use opioids trained in naloxone use	Interactive training process about overdose causes, prevention and IM naloxone administration Physician distributed naloxone	T_3m_: 10 individuals at follow up - 5/10 used naloxone successfully - 5/10 used other techniques successfully	CDC/NCIPC, NIH/CFAR, CDARR

US = United States, UK = United Kingdom, EMS = Emergency Medical Services, THN = Take-Home Naloxone.

**Table A3:** Summary of Included Studies from Professionals’ Perspectives.

Professionals’ Perspective						
Author, Title and Journal	Country	Data Collection Method	Participants	Interventions/Study Details	Outcomes	Funding/ Sponsorship
Ashrafioun et al., 2016 Evaluation of knowledge and confidence following opioid overdose prevention training: a comparison of types of training participants and naloxone administration method. Substance Abuse [75]	US	Assessment: T_0_: Baseline before training T_a_: Immediately after training	N = 93 medical providers N = 40 family and friends N = 3 patients N = 139 other	Training was 20–45 min on opioid overdose epidemiology, physiology and risk factors, naloxone’s role in overdose, its outcomes and how to use it both IM and IN	- All groups knowledge 83% increase and confidence 85% from pre-training (M = 4.2, SD = 1.1 and M3.7, SD 1.8) to post-(M = 6.2, SD 1 and M = 5.9, SD = 1.2) - Confidence and knowledge did not vary by route of administration - No difference in pre- or post-knowledge scores for participant type	Office of Academic Affiliations, Advanced Fellowship Program in the Mental Illness Research and Treatment, Department of Veterans Affairs
Bailey and Wermeling 2014 Naloxone for opioid overdose prevention: pharmacists’ role in community-based practice settings. Annals of Pharmacotherapy [64]	US	Interviews	N = 6 pharmacists	Participants from community and clinic-based settings were identified through their affiliations with collaborating providers	- Providers found a need for a prevention strategy for patients that they see on a day-to-day basis - Many pharmacies have developed their own educational protocols for patients - Barriers: financial and reimbursement, state regulations	National Center for Research Resources and the National Center for Advancing Translational Sciences
Behar et al., 2017 Acceptability of naloxone co-prescription among primary care providers treating patients on long-term opioid therapy for pain. Journal of General Internal Medicine [65]	US	Cross-sectional survey	N = 111 safety-net primary care prescribers who initiated naloxone co-prescribing 50% had been practicing for less than 5 years, most say 10 or less patients on opioids per month	Anonymous survey took five minutes to complete Physicians recruited via email	- 79.3% had co-prescribed naloxone since program initiation - 99.1% reported they were likely to co-prescribe naloxone - 3.6% suggested naloxone prescribing may lead to increase in opioid prescribing - Most frequent concerns around patient education	National Institutes of Health
Beletsky et al., 2007 Physicians’ knowledge of and willingness to prescribe naloxone to reverse accidental opiate overdose: challenges and opportunities. Journal of Urban Health [92]	US	Cross-sectional survey	N = 563 physicians 70% males, 83% white	Recruited from American Medical Association Physicians were mailed the survey and followed up with a phone call The survey assessed attitudes towards injecting drug users	- 129/563 (23%) had heard of prescribing naloxone as a prevention measure - 54% indicated that they would never “consider prescribing naloxone and explaining its use to an IDU patient” - Mean age of 51 rather than 48 was associated with having heard of this intervention	Substance Abuse Policy Research Program of the Robert Wood Johnson Foundation
Coffin et al., 2003 Preliminary evidence of healthcare provider support for naloxone prescription as overdose fatality prevention strategy in New York City. Journal of Urban Health [96]	US	Cross-sectional survey	N = 363 physicians 54% male, 68.4% white, 68% MD and 15.6% NP, 39.6% practice in an office and 32.1% in a hospital	Participants recruited from a list of prescribers obtained from the New York Department of Education 7–10 min survey measured attitudes around injecting drug users	- 33.4% of respondents reported that they would consider prescribing naloxone to patients at risk of opiate overdose; 29.4% were unsure, and 37.1% would not	None stated
Dahlem et al., 2016 Development and implementation of intranasal naloxone opioid overdose response protocol at a homeless health clinic. Journal of the American Association of Nurse Practitioners [76]	US	Intervention and post-survey: T_a_: Immediately after training	N = 35 homeless shelter staff	Overdose education and naloxone distribution flow chart was modified for non-healthcare staff and again for healthcare staff 1–2 h training in assembly and administration of IN naloxone, both didactic and hands on	T_a_: Training was reported average to excellent (mean score 4.54/5) - All learning outcomes were met - Participants suggested training be annually (19/35, 54%) or biannually (15/35, 43%)	None stated
Drainoni et al., 2016 Why is it so hard to implement change? A qualitative examination of barriers and facilitators to distribution of naloxone for overdose prevention in a safety net environment. BMC Research Notes [66]	US	Interviews and focus groups	N = 19 physicians N = 26 registered nurses N = 3 HPS N = 2 pharmacists 56% female, 34% over 25, 32% 2–5 years working in ED	All participants recruited from the ED department Interviews from 25–36 min, about barriers to new policy to increase naloxone distribution in emergency department	- Facilitators included: the intervention being real-world driven with leadership support, education and training efforts, availability of resources and ability to leave the ED with the naloxone kit in hand - Barriers: protocols and policy-related barriers, workflow and logistics, patient-related (stigma), staff roles and responsibilities education and training	Established Investigator Innovation Award from the Boston University School of Public Health
Gatewood et al., 2016 Academic physicians’ and medical students’ perceived barriers toward bystander administered naloxone as an overdose prevention strategy. Addictive Behaviors [67]	US	Interviews and discussion groups	N = 5 physicians N = 25 medical students	Participants were recruited due to their affiliation with 2 Baltimore medical schools Interviews and discussion groups were tailored to physician or student	- Barriers in prescribing naloxone to bystanders: duration of action of naloxone, route of administration, lack of knowledge about safety outside hospital setting, little basis for patient follow up, insult patients whom are not “addicted” but are at risk, “enabling” addicts to use more - Withdrawal symptoms, stigma associated with carrying naloxone	None stated
Green et al., 2013 Barriers to medical provider support for prescription naloxone as overdose antidote for lay responders. Substance use and misuse [68]	US	Qualitative interviews	N = 4 emergency department providers N = 9 substance use treatment providers N = 6 pain specialists N = 5 family medicine practitioners including doctors, nurses, and a physician assistant	Interviewees were nominated by member of the studies advisory board and then interviewees could further recommend participants	- Barriers: “Safety Net” effect allowing for risker drug use, the need to train the “Right People” with proper education and not a stand-alone solution	Centers for Disease Control and Prevention
Leece et al., 2015 Can naloxone prescription and overdose training for opioid users work in family practice? Perspectives of family physicians. Canadian Family Physician [69]	Canada	Focus groups and workshops	N = 17 family physicians	Two hour workshops and focus groups with physicians attending College of Family Physicians of Canada conference Focus groups performed a SWOT analysis	- Facilitators: safety, setting, engagement and education, logistics, and evidence - Barriers: guidelines and implementation, medicolegal uncertainties, support, equity or stigma, and evidence	None stated
Nielsen et al., 2016 Community pharmacist knowledge, attitudes and confidence regarding naloxone for overdose reversal. Addiction [70]	Australia	Cross-sectional survey	N = 595 pharmacists Average time practicing 6.5 years, 54.5% male, 51.1% aged 25–34	Random sample of pharmacies generated from an existing list of Australian community pharmacies and surveys were administered online	- Mean attitude score 6.18, reflecting positive attitudes toward harm-reduction services - 190/595 (31.9%) were confident in educating about naloxone use - Lowest levels of comfort reported in supplying naloxone to people buying syringes (68.4%) and opioid substitution therapy (77.6%) - Greater support for overdose was associated with whether the pharmacy provided OST (*p* = 0.004) -136/595 (23%) pharmacies stored naloxone - Mean score in naloxone knowledge test 1.8/5 - Barriers included time involved, lack of training and knowledge of state laws and lack of reimbursement for patient education and counselling	Substance Misuse Prevention and Service Improvements Grant
Sondhi et al., 2016 Stakeholder perceptions and operational barriers in the training and distribution of take-home naloxone within prisons in England. Harm Reduction Journal [71]	UK	Qualitative interviews	N = 17 key stakeholders N = 26 prisoners	Both formal, structured and informal discussions with a range of stakeholders including prison representatives from healthcare and substance misuse services, strategic leads for NHS England and representatives from Martindale Pharma Four focus groups of male prisoners about views on participating in training	- Prisoners were sometimes confused about the message of THN as most primary prison goals were to abstain from opioid use - Staff found a disconnect between THN as harm reduction and potential incentive for using drugs - Staff had concerns about who and when to give THN training, the logistical issues of distributing THN within a prison setting and the need for a “whole systems” approach, involving senior prison staff.	National Health Service England
Tobin et al., 2005 Attitudes of emergency medical service providers towards naloxone distribution programs. Journal of Urban Health [72]	US	Cross-sectional survey	N = 154 emergency medical service providers 79% male, 75% white, mean age 34	Surveys were distributed to EMS providers who attended an educational class	- 74% had heard of a naloxone program - 84/154 (56%) did not view naloxone training as effective in reducing deaths - 96/154 (64%) reported no interest in participating in training drug users; however, 47/154 (30%) reported interest in a class about addiction and health issues education - Barriers: dirty needles, naloxone theft, administration and other acute health factors were raised	National Institute on Drug Abuse
Winstanley et al., 2016 Barriers to implementation of opioid overdose prevention programs in Ohio. Substance Abuse [73]	US	Cross-sectional survey with quantitative and qualitative questions	N = 20 OOPP site representatives 47.8% were in suburban countries, 34.8% in urban countries and 17.4% in rural communities	Contact person of opioid overdose prevention programs (OOPP) were given a survey about their program Initial list from the Ohio Department of Health Survey was emailed or given over the phone	- 15/20 (83%) of programs experience barriers, which were categorized into: stigma (n = 14), costs (n = 7), staffing (n = 5), legal (n = 4), regulatory (n = 3) and clients (n = 3).	None stated
Zaller et al., 2013 The feasibility of pharmacy-based naloxone distribution interventions: a qualitative study with injection drug users and pharmacy staff in Rhode Island. Substance Use and Misuse [74]	US	Qualitative interviews	N = 21 pharmacy staff Median age 35, 80% white, 73.3% male N = 21 people who inject drugs Median age 36 and 71% white, 66% male	People who inject drugs were recruited from syringe exchange programs and recognized community locations Pharmacy staff were recruited through in-person visits or phone calls Interviews lasted 45 min to 1.5 h	- Pharmacists were not aware of the prevalence of overdose and all IDUs reported experiencing or witnessing an overdose - Positive and negative attitudes toward naloxone use from injecting drug users - Pharmacy staff were concerned about increase in risk behaviours - Overall, many IDUs and pharmacy staff were supportive - Both groups misjudged each other’s willingness to participate in a naloxone intervention	None stated

ED = Emergency Department, EMS = Emergency Medical Services, IM = Intramuscular, IN = Intranasal, IDU/s = Injecting Drug Use/Users, MD = Medical doctor, NHS = National Health Service, NP = Nurse Practitioner, OOPP = Opioid Overdose Prevention Programs, OST = Opioid Substitution Therapy, SWOT = Strength Weakness Opportunities Threats, THN = Take-Home Naloxone, UK = United Kingdom, US = United States.
